# Supplementary material for: 3D printing colloidal crystal microstructures via sacrificial-scaffold-mediated two-photon lithography
Source: Nat Commun. 2022 Aug 5;13:4563. doi: 10.1038/s41467-022-32317-w (PMC9355982; doi:10.1038/s41467-022-32317-w)
Supplement: Supplementary file 1 — Supplementary information [file 41467_2022_32317_MOESM1_ESM.pdf]

Supplementary information

3D Printing Colloidal Crystal Microstructures via Sacrificial-scaffold-mediated Two-photon Lithography

Keliang Liu[1], Haibo Ding[1], Sen Li[1], Yanfang Niu[1], Yi Zeng[1], Junning Zhang[1], Xin Du[1*] & Zhongze Gu[1*]

Experimental Section

Materials: bis(2-methacryloyl)oxyethyl disulfide (BMOD, 99%), poly(ethylene glycol)diacrylate (PEGDA, Mw = 700, 99%), N-isopropylacrylamide (NIPAAm, 99%), tris(2-carboxyethyl)phosphine (TCEP, 98%) and 2-hydroxy-2-methylpropiophenone (HMPP, 97%) were purchased from Sigma-Aldrich. N,N'-methylenebisacrylamide (Bis, 99%), acrylic amide (AAm, 99%), 2-Hydroxyethyl methacrylate (HEMA, 99%), ethylene dimethacrylate (EDMA, 98%), pentaerythritol tetraacrylate (PTTA, 80%), 2-hydroxy-4-(2-hydroxyethoxy)-2-methylpropiophenone (Irgacure 2959, 98%), diphenyl(2,4,6-trimethylbenzoyl)-phosphine oxide (TPO, 97%), ethylene glycol, N,N-dimethylformamide (DMF, 99%) and hydrofluoric acid (HF, 40%) were purchased from Aladdin (China). Gelatin methacrylate (GelMA, 95%) were purchased from AvaTarget (China). Monodispersed silica nanoparticles (Φ = 150 & 180 nm) were purchased from Nano rainbow (China).

Preparation of degradable colloidal crystal hydrogel (sacrificial scaffold): silica nanoparticles were dispersed in DMF (65 wt.%) and sonicated for 30 minutes. Then 4 μL BMOD, 0.1 mg photo initiator 2959 and 50 μL of the above solution were mixed, followed by sonicate for 15 minutes. The mixture was filled into a gap between two parallel glass substrates separated by a polyimide spacer (75 μm thickness) and exposed to UV (70 mW/cm2 at 365 nm) for 5 min. A degradable colloidal crystal hydrogel film with bright structure color was obtained.

Preparation of precursor solutions for secondary polymerization: 0.8 mL PEGDA, 0.2

mL deionized water and 8 mg TPO were mixed, followed by stirring for 30 min to obtain the PEGDA precursor solution. Composition of other precursor solutions are listed as follows:

NIPAAm precursor: 300 mg NIPAAm, 20 mg Bis and 5 mg TPO in 1 mL ethylene glycol.

AAm precursor: 250 mg AAm, 10 mg Bis and 5 mg 2959 in 1 mL deionized water.

HEMA precursor: 0.97 mL HEMA, 0.03 mL EDMA and 10 μL HMPP.

GelMA precursor: 100 mg GelMA, 10 mg 2959 in 1 mL deionized water.

PTTA precursor: pure PTTA monomer containing 10 mg/mL 2959.

TPL fabrication in sacrificial scaffold: the precursor solution was first loaded into the sacrificial scaffold via solvent exchange. To do this, the sacrificial scaffold with glass substrate was immersed in the precursor solution for 24 hours to ensure the complete substitution. Then the sacrificial scaffold was placed into a commercial two-photon direct writing system (Nanoscribe GT+, Germany) with a 25× objective (numerical aperture of 0.8 from Zeiss). The hatching and slicing of all microstructures in the experiment are 0.3 μm and 0.5 μm, respectively. In the TPL process, the relative movement of the laser focus in the sacrificial scaffold is achieved by controlling the movement of Galva-nometric scanners and high-resolution x/y/z stages. And the adjustment of laser power and scanning speed can be modified by nanowrite software. During this process, secondary polymerization is induced by the high-energy laser in the degradable scaffold, while the arrangement of the nanoparticles is "locked" by the highly cross-linked degradable network. In this way, micro hydrogels with presetted geometries can be generated in the sacrificial scaffold by TPL without disturbing the ordered assembly of the inner nanoparticles.

.

Degradation of sacrificial scaffold: after TPL processing, the sacrificial scaffold (on glass substrate) is degraded by immersing in a solution of 0.1 mol/L TCEP in DMF for

1h. The sacrificial scaffold and unpolymerized precursor were removed in this process, leaving the TPL-fabricated microstructures with orderly-arranged inner nanoparticles (attached on glass substrate).

Fabrication of inverse opal microstructures: the degradable colloidal crystal hydrogel film was prepared on zirconia substrate. Next, the hydrogel was infused with the PTTA precursor in the dark. Then, TPL was performed, and microstructures were obtained after degradation. The microstructures were placed in 5 wt% hydrofluoric acid aqueous solution for 12 hours, which etches silica nanoparticles to obtain inverse opal microstructures.

Optical images acquisition: for macroscopic colloidal crystal films, the optical images are taken by a Sony A6300 digital camera. For colloidal crystal microstructures, all optical images are taken under a stereo microscope (Mshot MJ30), and the light environment is provided by the built-in light source of the microscope. For some images, a black cardboard was placed under the glass substrate to make the structure color of the micro-objects more clearly. In this paper, without special declaration, all structural color microstructure images were taken in DMF solution environment. All optical images of thermosensitive (polyNIPAAm) microhydrogels were taken in deionized water environment.

Reflective spectra measurement: the reflective spectra of the colloidal crystal materials in this paper are acquired using the microscope external fiber optic spectrometer (Ocean Optics, QE65000). The position of the microstructures was first determined under microscope, then the corresponding reflective spectrum was obtained using the spectrometer.

Extraction of color information from structural color microstructures: to obtained the hue information, we imported the obtained optical images into MALAB and switched

color space of the images from RGB to HSV. Then the hue vale of the selected pixels can be obtained directly. In this process, a technical problem will occur, that the red and purple are connected on the hue ring and look similar, during the imaging process, the camera may mistakenly records red pixels as purple pixels, thus the hue mapping results at red area would be highly rough. To solve this problem, we removed all pixels with hue value larger than 0.95 to obtain a relatively accurate average hue within the data range. In addition, the Gaussian filter function was used to smooth the noise generated by the CCD camera in the hue map.

SEM analysis: SEM images were obtained using a field emission scanning electron microscope (Zeiss Ultra Plus, Germany). The microstructures were freeze-dried by vacuum freeze dryer (BIOCOOL, China) and sputtered with 30 nm gold layer using a Hitachi E-1010 ion sputter (Hitachi, Ltd., Japan) before measurement.

Raman spectrometry: Raman spectra of different hydrogel films were obtained by Raman spectroscopy (Renishaw invia 2jgy39). The hydrogel films were dehydrated prior to testing. The test laser wavelength was chosen to be 532 nm with a laser energy of 50%. Due to the weak intensity of the Raman peak of the disulfide bond, the integration time is 10 seconds and the number of integrations is 4 times.

Temperature sensing experiment: the temperature-sensitive microsensor was fabricated on a glass substrate, which is then encapsulated by a PDMS cover to form a microfluidic chip. During the experiment, the microsensor was completely immersed in water, and the ambient temperature of the microsensor can be changed by pumping water with different temperatures into the microfluidic channel.

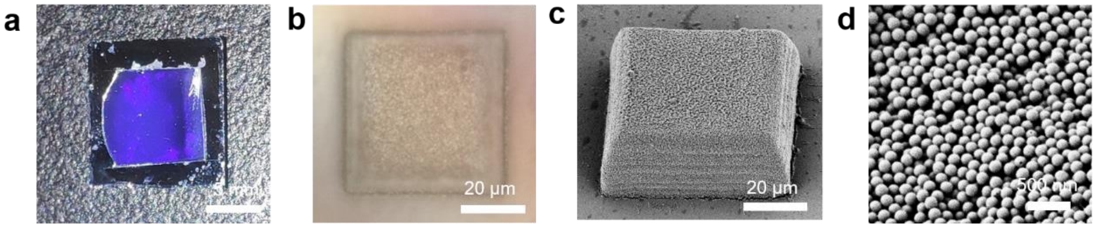

Supplementary Fig. 1 The demonstration of the macroscopic colloidal crystal hydrogel film and the microhydrogel fabricated with nanoparticle-containing PEGDA precursor solution. **a** Photo of a colloidal crystal hydrogel film fabricated by UV illuminating a nanoparticle-containing PEGDA precursor solution. A bright blue structure color could be seen. **b** An example of the microhydrogel fabricated by direct TPL in the same nanoparticle-containing PEGDA precursor solution. No structure color was observed. We fabricated microhydrogels using precursors with different PEGDA concentrations and with different processing parameters, it’s rather difficult to obtain a hydrogel cube with even tiny structure color effect. **c** SEM image of the fabricated colloidal crystal microstructure. **d** Magnified SEM image indicates that the ordered arrangement of the nanoparticles in the hydrogel is interrupted. Scale bar: 500 nm.

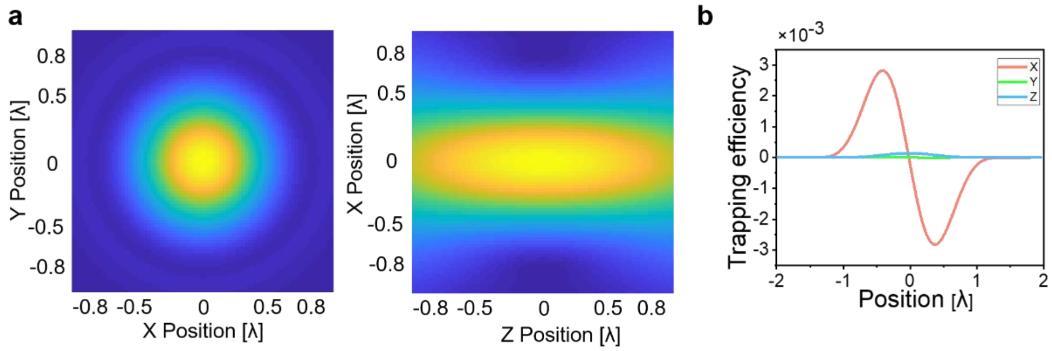

Supplementary Fig. 2 Simulation calculation of optical field gradient force. **a** The light field distribution map of Gaussian beam (λ_{fs} = 780 nm). **b** The relationship between the trapping efficiency and the displacement from beam focus.

The trapping efficiency is calculated as follows:

The optical tweezers computational toolbox[1] is employed to construct the optical field distribution of Gaussian beam and the T-matrix of the contained nanoparticle optical information through MATLAB. The Gaussian beam is circularly polarized by default, the laser wavelength is 780 nm and the laser power (*P*) is 20 mW. The refractive index of the environmental medium is 1.427. The numerical aperture (NA) input is 0.8. The nanoparticles used to construct the T-matrix are silica particles with a particle diameter of 150 nm, with a refractive index of 1.45. The trapping efficiency we calculated is a dimensionless value. When it comes to actual forces, we use the formula

$$F = \frac{np}{c} Q \tag{1}$$

Where *n* is the refractive index of the environmental medium, *P* is the laser power and *c* is the speed of light in vacuum. *Q* is trapping efficiency. Calculated from the above formula, the maximum optical field gradient force (in x, y axis) along the laser scanning direction is about 2.85×10^{-2} pN, which can perturb the ordered arrangement silica particles.

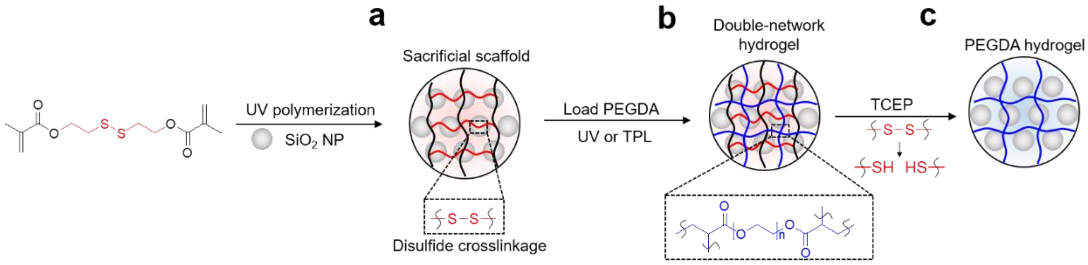

Supplementary Fig. 3 Schematic demonstration of the sacrificial-scaffold-mediated TPL process. **a** Formation of the sacrificial scaffold. **b** Secondary polymerization inside sacrificial scaffold, forming double-network hydrogel. **c** Degradation process, removing the sacrificial scaffold, leaving the secondary polymerized hydrogel network with orderly-arranged SiO_2 nanoparticles inside.

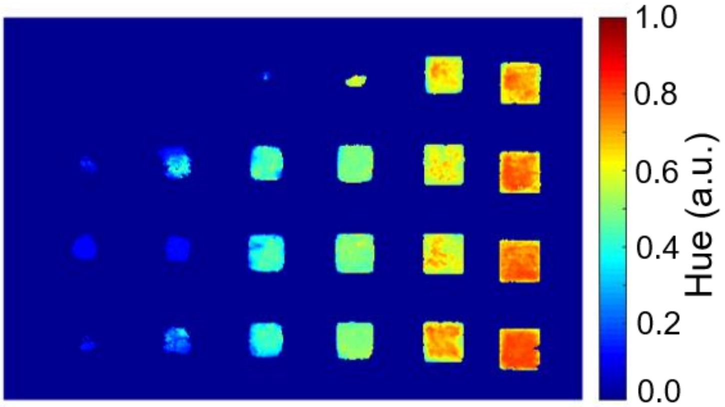

Supplementary Fig. 4 Hue map of the microcube array shown in Fig. 3d. The hue values are extracted with MATLAB.

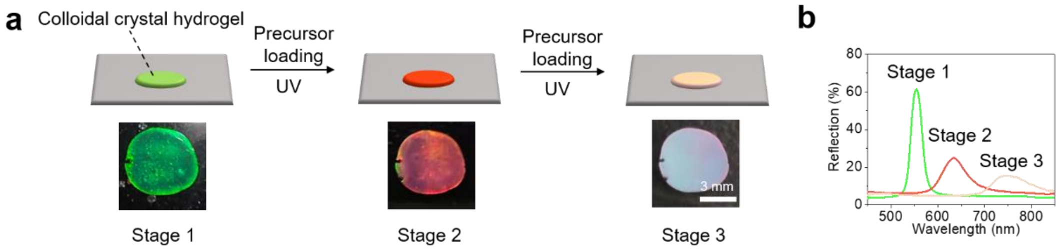

Supplementary Fig. 5 Effect of the solid content of the colloidal crystal hydrogel film on its revealed structure color. **a** Schematic demonstration of the fabrication of hydrogel films with different solid content, and photo of the generated hydrogel films. **b** Reflective spectra of the hydrogels in **a**.

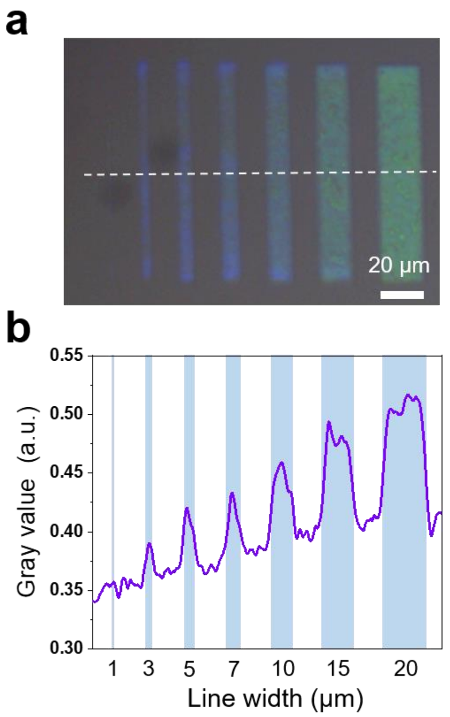

Supplementary Fig. 6 Resolution test. Hydrogel lines with 1, 3, 5, 7, 10, 15 and 20 μm width were fabricated in a sacrificial scaffold loaded with PEGDA precursor. The results show that the smallest line that can be fabricated is 3 μm. **a** Photo of the fabricated hydrogel lines. **b** Change of the gray value along the dashline in **a**, indicates the existence of the fabricated hydrogel lines. The grey value data can be obtained through ImageJ.

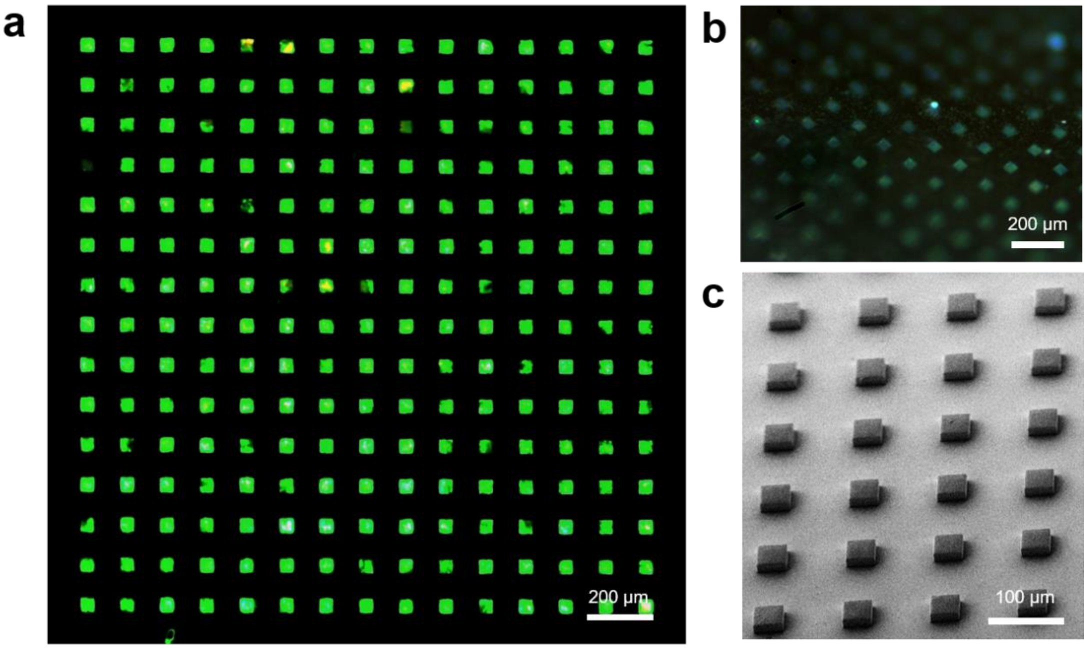

Supplementary Fig. 7 Images from reproducibility test. **a** Photo (top view) of the fabricated 225 hydrogel microcube (40×40×20 μm), the cubes exhibit uniform structure colors with average Hue value of 0.327 ± 0.03. **b** Side view of the cube array. **c** SEM image of the freeze-dried hydrogel cube array, indicating the uniform geometry of the obtained cubes. The images demonstrate that our method is highly reproducible.

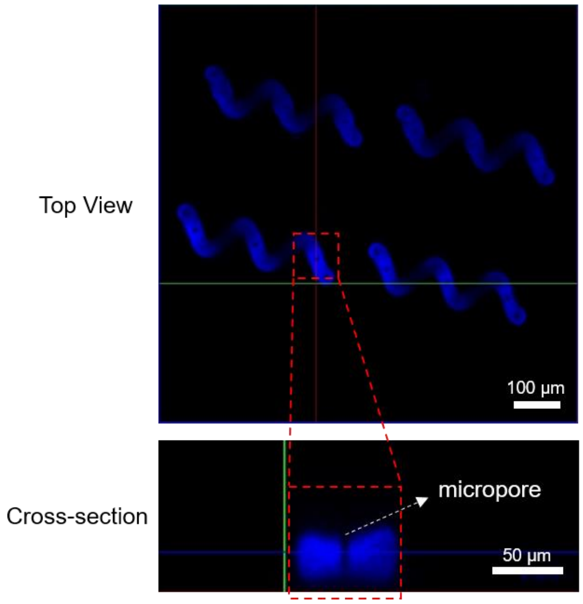

Supplementary Fig. 8 Laser scanning confocal microscopy test on the fabricated 3D hollow spiral hydrogel micropipe. The hydrogel exhibits blue fluorescence, thus confocal microscopy was utilized to investigated the 3D structure of the microhydrogel. The bottom image shows the cross-section view of the micropore, indicating that the micropore is completely through.

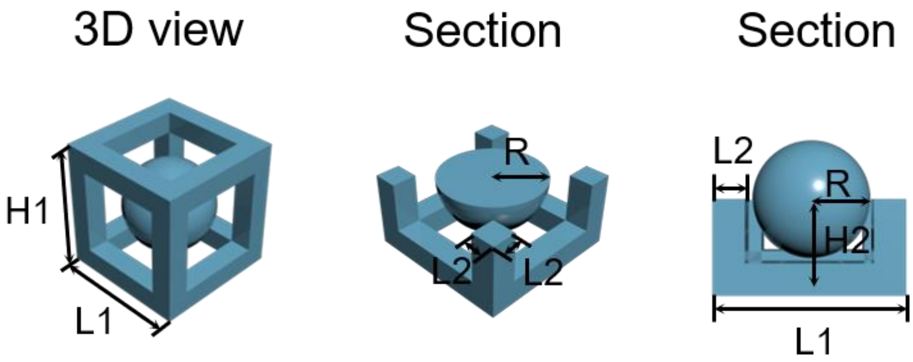

Supplementary Fig. 9 Structure of the designed suspended structure. H1 = L1 = 60 μm, L2 = 10 μm, R = 22 μm, H2 = 30 μm.

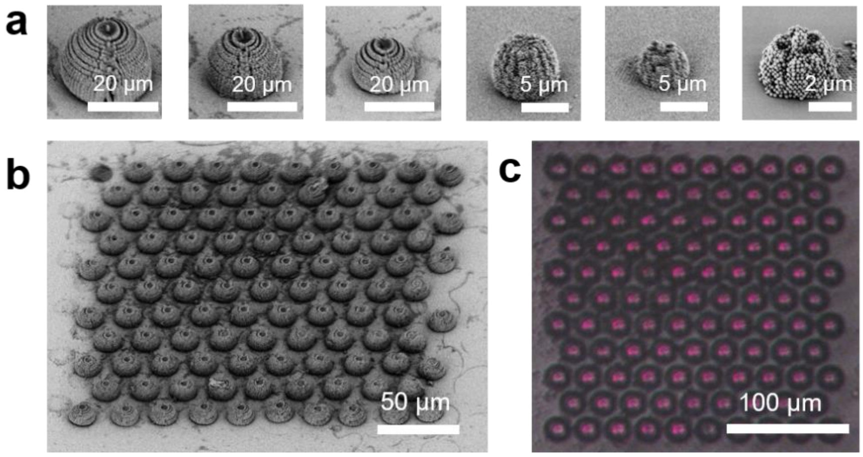

Supplementary Fig. 10 **a** The SEM image of freeze-dried hierarchical colloidal particles with different feature sizes. The smallest hierarchical particle that can be generated is with the diameter of ~ 4 μm. **b** The SEM image and **c** the optical image of a hierarchical colloidal particle array in hexagonal close-packed state.

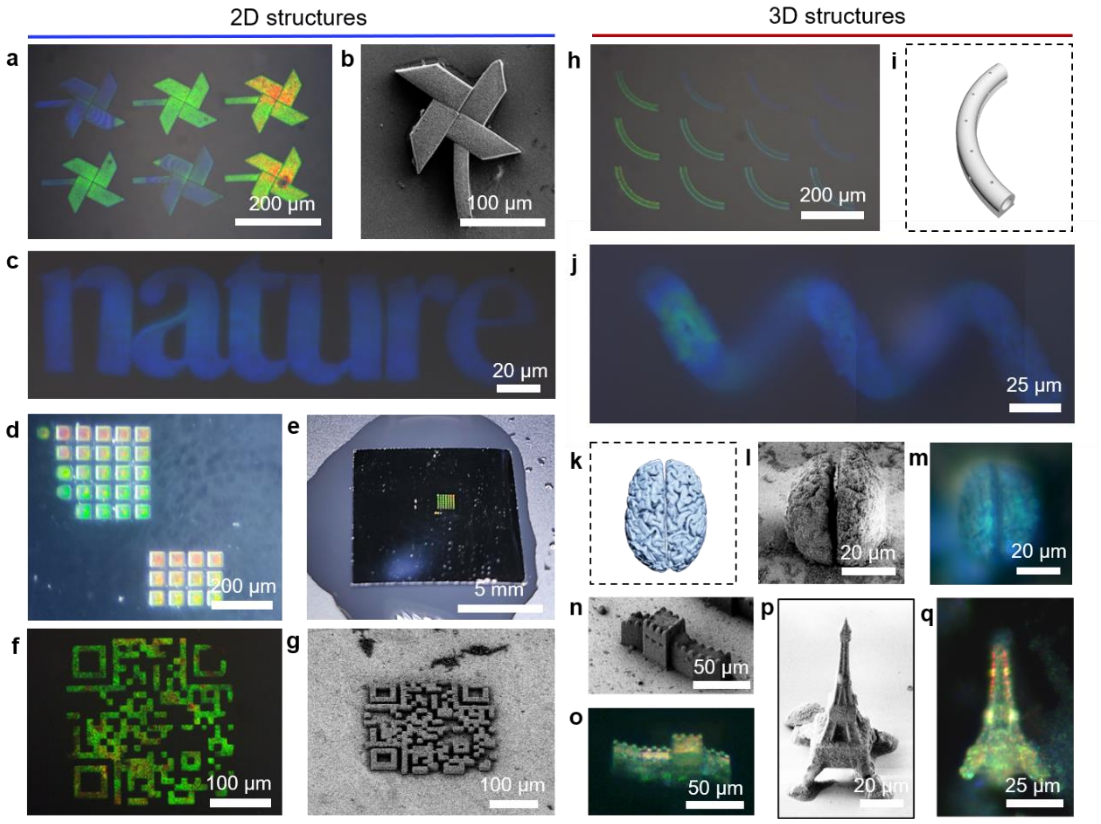

Supplementary Fig. 11 Various 2D and 3D microhydrogels fabricated with the sacrificial-scaffold-mediated TPL. **a-g** Photo and SEM images of different 2D microhydrogels. **h-q** Design, photo and SEM images of different 3D microhydrogels. **j** is the microscopic image of the hollow spiral hydrogel micropipe, generated by the merge of 3 photos.

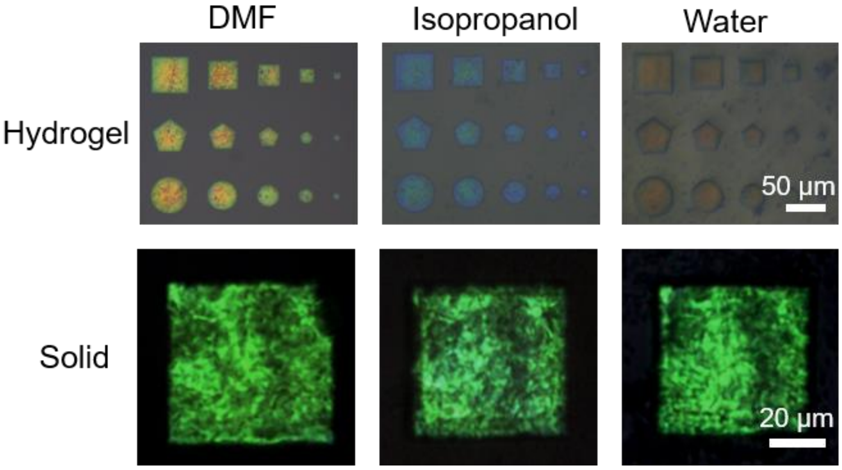

Supplementary Fig. 12 Photo of the fabricated hydrogel-state and solid-state colloidal crystal microstructures under DMF, isopropanol and water. The structure color of the microhydrogels varied upon the solvents, while the solid-state colloidal crystal microcube kept constant color in different solvent.

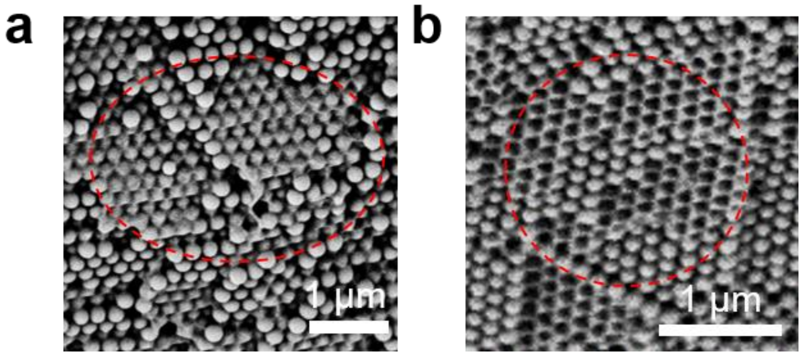

Supplementary Fig. 13 SEM image of the fabricated **a** hydrogel-state and **b** solid-state colloidal crystal microstructures. In microhydrogel, after freeze-dried, the hydrogel network collapsed to form polymer films on part of the nanoparticles. While in solid-state microstructure, such phenomenon does not appear. Instead, clear inverse opal structure was observed in this case, indicating that the gap between nanoparticles are filled with solid polymer network.

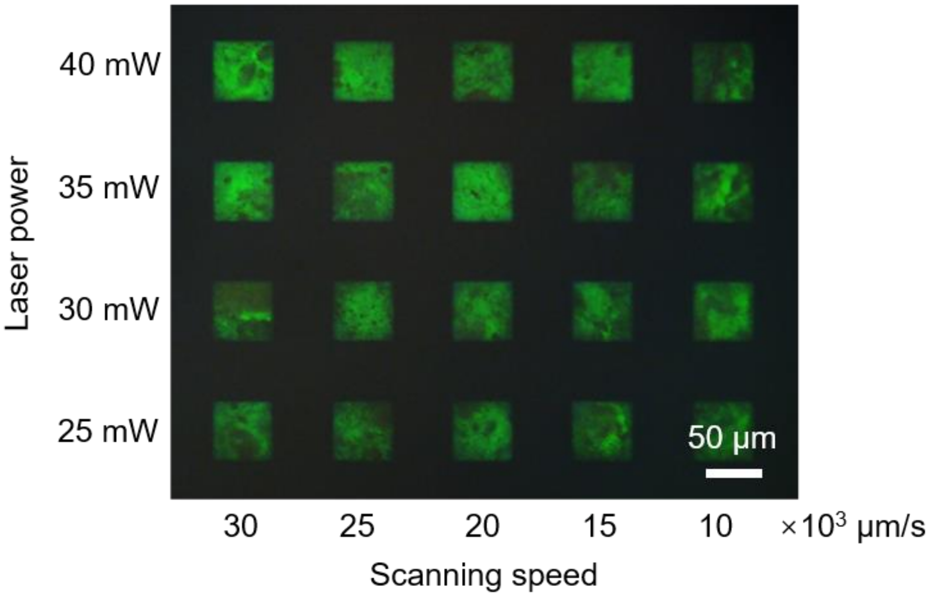

Supplementary Fig. 14 Solid-state colloidal crystal microcube array fabricated with different TPL processing parameters. All cube exhibit same green structure color, indicating that the TPL processing parameter does not affect the color of the obtained colloidal crystal materials.

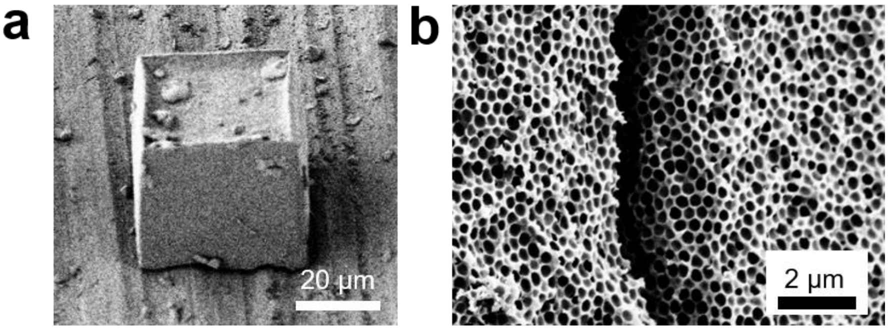

Supplementary Fig. 15 The demonstration of the inverse opal microstructure. **a** The SEM image of the inverse opal microstructure adhered to zirconia substrate. **b** The morphology of residual cross-linked network.

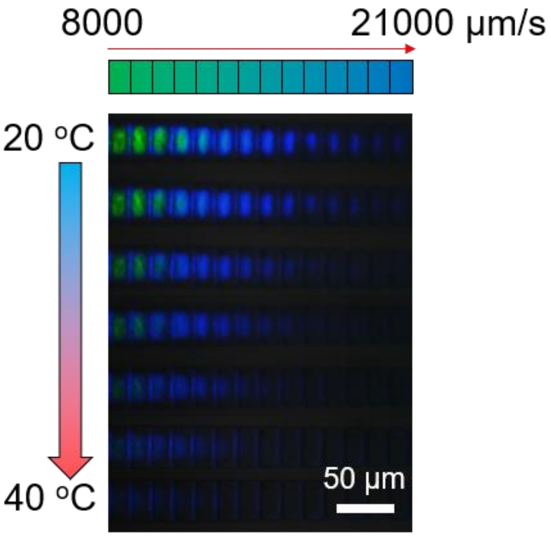

Supplementary Fig. 16 The temperature "tag" fabricated using NIPAAm precursor. The laser power during TPL fabrication is 20 mW. Scale bar: 30 μm.
